# Repeat community-based screening for school-age congenital heart disease: impact on the detection of cases requiring treatment in resource-limited settings

**DOI:** 10.1007/s12519-026-01060-3

**Published:** 2026-07-27

**Authors:** Kai Bai, Shu-Yue Zhang, Teng Wang, Yan Wang, Yue Gao, Feng-Shu-Yue Li, Tao Wang, Miao Zhao, Wei Luo, Yu-Ling Lu, Li-Ping He, Jiang Lu, Yu Xia, Lin Duo

**Affiliations:** 1 Fuwai Yunnan Hospital, Chinese Academy of Medical Science, Affiliated Cardiovascular Hospital of Kunming Medical University, Kunming 650102, China; 2 School of Public Health, Kunming Medical University, Kunming 650500, China; 3 Yunnan Population and Family Planning Research Institute, No.146, Qingnian Road, Wuhua District, Kunming 650021, China

**Keywords:** Community screening, Missed diagnosis, Repeat screening, School-age children

## Abstract

**Background:**

School-based screening is a critical strategy for detecting congenital heart disease (CHD) in resource-limited regions, yet single-round programs may miss a substantial number of treatable cases. The value of repeat screening in identifying these missed cases remains underexplored.

**Methods:**

As part of the Yunnan Provincial Comprehensive Plan for CHD Prevention and Control, we conducted two rounds of community-based CHD screening using cardiac auscultation followed by portable echocardiography in 23 counties. The first round (2017–2020) screened 1,024,998 children aged 3–18 years, and the second round (2021–2025) screened 1,059,156 children after intervals of 4, 5, 6, or 7 years. Children with CHD requiring treatment were identified and categorized by screening history.

**Results:**

The first screening detected 938 cases (prevalence: 0.92/1000), while the second screening detected 557 cases (prevalence: 0.53/1000). Of note, a significant proportion of cases identified in the second round represented missed diagnoses from the first screening. When comparing these missed cases (“previously covered”) to the true new cases identified in the second round (“newly covered”), the “newly covered” cases were younger and had lower odds of moderate or great anatomical complexity [adjusted odds ratio (OR) for moderate complexity: 0.58, 95% confidence interval (CI) = 0.36–0.92; adjusted OR for great complexity: 0.29, 95% CI = 0.11–0.71].

**Conclusions:**

Repeat school-based screening identifies a distinct group of previously missed and newly covered CHD cases, many of which are of higher anatomical complexity and would otherwise remain undiagnosed. These findings underscore the necessity for periodic rescreening to mitigate diagnostic gaps and improve CHD outcomes in resource-limited populations.

**Graphical Abstract:**

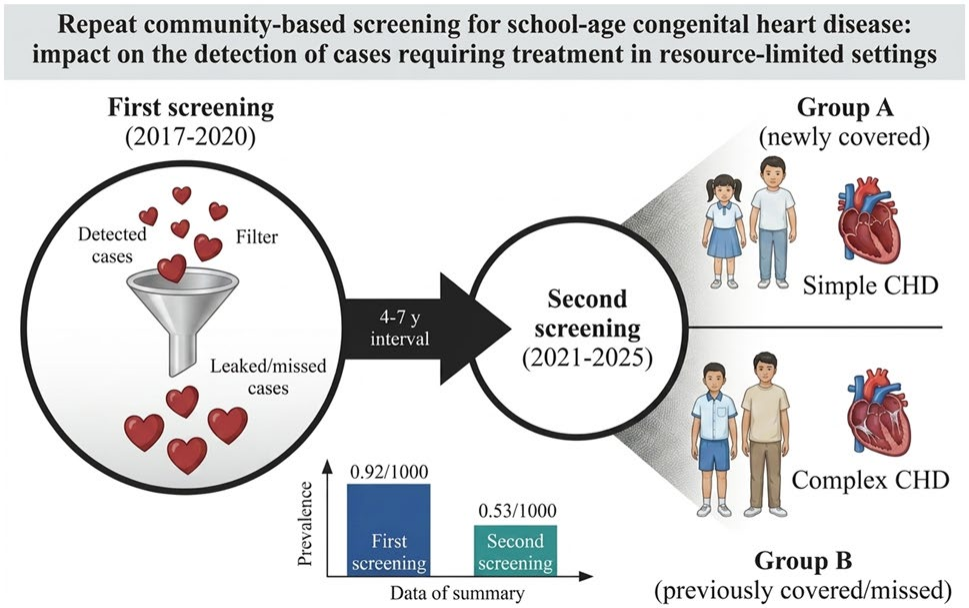

**Supplementary Information:**

The online version contains supplementary material available at 10.1007/s12519-026-01060-3.

## Introduction

Congenital heart disease (CHD) is the most common category of major congenital anomalies worldwide [1], representing nearly one-third of all such conditions and affecting an estimated 12 million individuals across all ages as of 2017 [2, 3]. The global birth prevalence of CHD was estimated at 9.41 per 1000 live births in 2010–2017, but substantial geographical disparities exist [4]. While high-income regions have seen marked improvements in survival and long-term outcomes due to advanced diagnostics and care, the burden has remained disproportionately high in low- and middle-income countries [2, 5]. Moreover, recent meta-analyses indicated that the reported birth prevalence of CHD in low-income settings, particularly in Africa, was consistently underestimated due to limited access to prenatal and neonatal diagnostic resources [6]. This diagnostic gap resulted in a significant “hidden” population of undiagnosed cases in the community. To address this unmet need, school-based CHD epidemiological screening has emerged as a crucial complementary strategy for identifying cases of affected individuals [7, 8]. There is a pressing need for epidemiological studies of CHD among school-age children [9, 10].

The reported prevalence of CHD among school-age children varies widely across populations. A report from Nigeria indicated a considerably higher rate of 15 per 1000 [11]. In contrast, a study from India reported a lower prevalence of 0.46 per 1000 [12]. Notable regional studies include reports of 5.66 per 1000 in the Qinghai-Tibetan Plateau of China [9], 6.94 per 1000 in a Yunnan cluster-sampling study [10], and 13.0 per 1000 in an echocardiographic screening of over 21,000 children in rural China [13]. Despite the recognized value of school-age screening, existing data were predominantly cross-sectional. Current multi-year surveillance in most regions is confined to passive, hospital-based systems focused primarily on birth cohorts [14, 15]. Such single-time-point assessments cannot capture index cases over time and any possible CHD that was previously “missed”. This represents a significant gap that limits both epidemiological understanding and the development of effective, longitudinal public health strategies.

Crucially, while school-based screening programs have been implemented in various settings, a proportion of CHD cases requiring treatment remained undetected after initial screening due to the limitations of method sensitivity, evolving clinical presentation, and population mobility. The role of repeat screening in identifying these missed cases, particularly those with higher anatomical complexity that may have been overlooked at first screening, is a critical yet understudied aspect of CHD surveillance. To address this gap, this study aimed to evaluate the impact of repeat community-based screening on the detection of previously missed and index CHD cases, and to examine how different screening intervals influence the profile of identified cases in resource-limited settings. The findings of this study are intended to inform and strengthen CHD screening strategies in similar populations worldwide.

## Methods

### Study design and participants

This cross-sectional study was conducted as part of the Yunnan Provincial Comprehensive Plan for CHD Prevention and Control in Children (YCHD) by Fuwai Yunnan Hospital between 2017 and 2025. The program has covered 74.4% of counties/districts in Yunnan Province, targeting kindergarten, primary, and secondary school children. From these, 23 counties/districts were selected for repeated screening using identical protocols at varying intervals (4, 5, 6, or 7 years) within the same geographic areas. Data from these 23 counties/districts formed the basis of the present analysis.

The study population comprised all school-age children (3–18 years) enrolled in educational institutions within the selected screening regions; it was designed to be inclusive. All eligible children present during screening visits were enrolled, with rare exceptions (e.g., temporary absence from school on the screening day). The requirement for treatment was determined by on-site cardiologists and was based on the B-ultrasound screening result. Exclusion criteria included: (1) children with previously diagnosed CHD who had completed treatment; and (2) those with minor cardiac defects not requiring intervention, such as small patent foramen ovale, atrial septal defect (ASD) < 5 mm, or ventricular septal defect (VSD) < 3 mm in the absence of clinical symptoms.

#### Classification of children with congenital heart disease requiring treatment

Children identified as requiring treatment during the first (*n* = 938) and the second screening (*n* = 557) were analyzed separately. Those detected in the second screening were further classified in two ways: (1) by prior screening history: “previously covered” (children with CHD who had undergone the first screening round but were not detected until the second round) and “newly covered” (not age-eligible: children under three years old during the first round), with the important caveat that we lacked individual-level medical records or screening results from the first round, meaning this classification reflects geographic exposure rather than confirmed prior auscultation or a documented “healthy” status. Eligibility for the first screening was determined retrospectively based on date of birth and date of screening; (2) by interval between screening: 4, 5, 6, or 7 years. A schematic overview of these classifications is provided in Supplementary Fig. 1.

### Data collection

A standardized screening protocol was implemented across all sites. Each screening team consisted of one cardiologist (team leader), one qualified cardiac ultrasound physician, 8–10 paramedical staff who had undergone specialized training in cardiac auscultation, and 1–2 local volunteers. Two teams operated concurrently to ensure efficient coverage.

All participants first underwent a physical examination and cardiac auscultation conducted by trained paramedical staff. For cases with subtle murmurs or any uncertainty, a second evaluation was performed by an experienced cardiologist to confirm the auscultation findings. Following this, all suspected cases underwent echocardiography using a portable ultrasound system (Philips X50®) performed by a qualified cardiac ultrasound physician. Diagnoses were documented by trained volunteers according to the ICD-10-CM coding system (Supplementary Table 1) [16]. This multi-step process, carried out entirely by trained and experienced professionals, was designed to balance screening efficiency with diagnostic accuracy.

For children confirmed with CHD, the need for treatment was determined by cardiologists based on: (1) the presence of related symptoms (e.g., cryptogenic stroke, migraine, dyspnea); (2) objective evidence of right ventricular volume overload; or (3) a clinically significant defect size (e.g., ASD ≥ 5 mm). Treatment decisions were confirmed by consensus of at least two senior cardiologists.

Cases requiring treatment were categorized into six pathophysiological groups: ASD, VSD, patent ductus arteriosus (PDA), combined CHD, complex CHD, and other CHD. Complex CHD included severe anomalies such as tetralogy of Fallot, hypoplastic left heart syndrome, and complete transposition of the great arteries. Combined CHD is cases with multiple major defects. Subtypes with fewer than ten cases were grouped as “other CHD”. Anatomical and clinical severity were classified using the American Heart Association (AHA) three-tier framework: simple, moderate complexity, and great complexity [17].

### Statistical analysis

Categorical variables were summarized as frequencies and percentages (%). Continuous variables were tested for normality using the Shapiro–Wilk test and Q–Q plots; non-normally distributed variables were reported as median and interquartile range. Between-group comparisons used Chi-square or Fisher’s exact tests for categorical variables, and Mann–Whitney *U* or Kruskal–Wallis tests for continuous variables.

Multinomial logistic regression models were used to assess the association between CHD anatomical complexity and three exposure variables: (1) screening stage (first vs. second screening); (2) screening interval (4, 5, 6, 7 years); (3) prior screening history (newly covered vs. previously covered), analyzed only within the second screening cohort. Each model was adjusted for age, sex, ethnicity, residential altitude, and regional type, with the simple complexity category as the reference. Analyses were performed using R software (version 4.4.3). A two-sided *P* value < 0.05 was considered statistically significant.

## Results

### Prevalence of congenital heart disease in 23 repeat screening counties requiring treatment

The first screening covered 1,024,998 children and identified 938 cases of CHD requiring treatment; a prevalence of 0.92 per 1000. The second screening examined 1,059,156 children and detected 557 cases; a prevalence of 0.53 per 1000. Cumulatively, 2,084,154 screenings were performed across both stages, identifying 1495 children with treatable CHD. The overall prevalence across the duplicate screening initiative was 0.72 per 1000 children (Table 1).

**Table 1:** Aggregated screening data and prevalence rates for children requiring treatment for congenital heart disease (CHD)

Screening stages	Number of counties	Number of townships/subdistricts	Number of schools screened	Number of children screened	Cases requiring treatment	Prevalence of CHD requiring treatment/1000
First screening	23	247	2645	1,024,998	938	0.92
Second screening	23	229	2692	1,059,156	557	0.53
Total	46	476	5337	2,084,154	1495	0.72

**Fig. 1:**
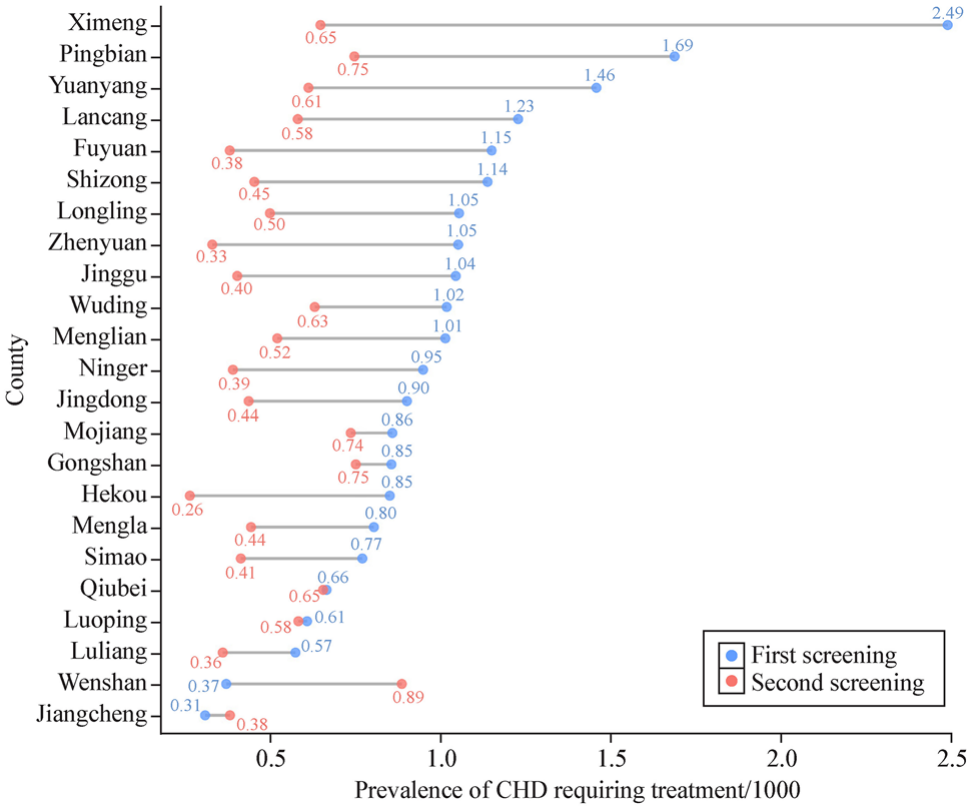
Regional prevalence of congenital heart disease (CHD) requiring treatment. Data show prevalence rates between the first and second screenings

Figure 1 shows that in 21 of the 23 counties with repeat screens, the prevalence of CHD requiring treatment was lower during the second screening round. The only two counties where second-round prevalence exceeded first-round rates were Wenshan and Jiangcheng.

### Characteristics of children with congenital heart disease requiring treatment by screening stage and screening interval

**Fig. 2:**
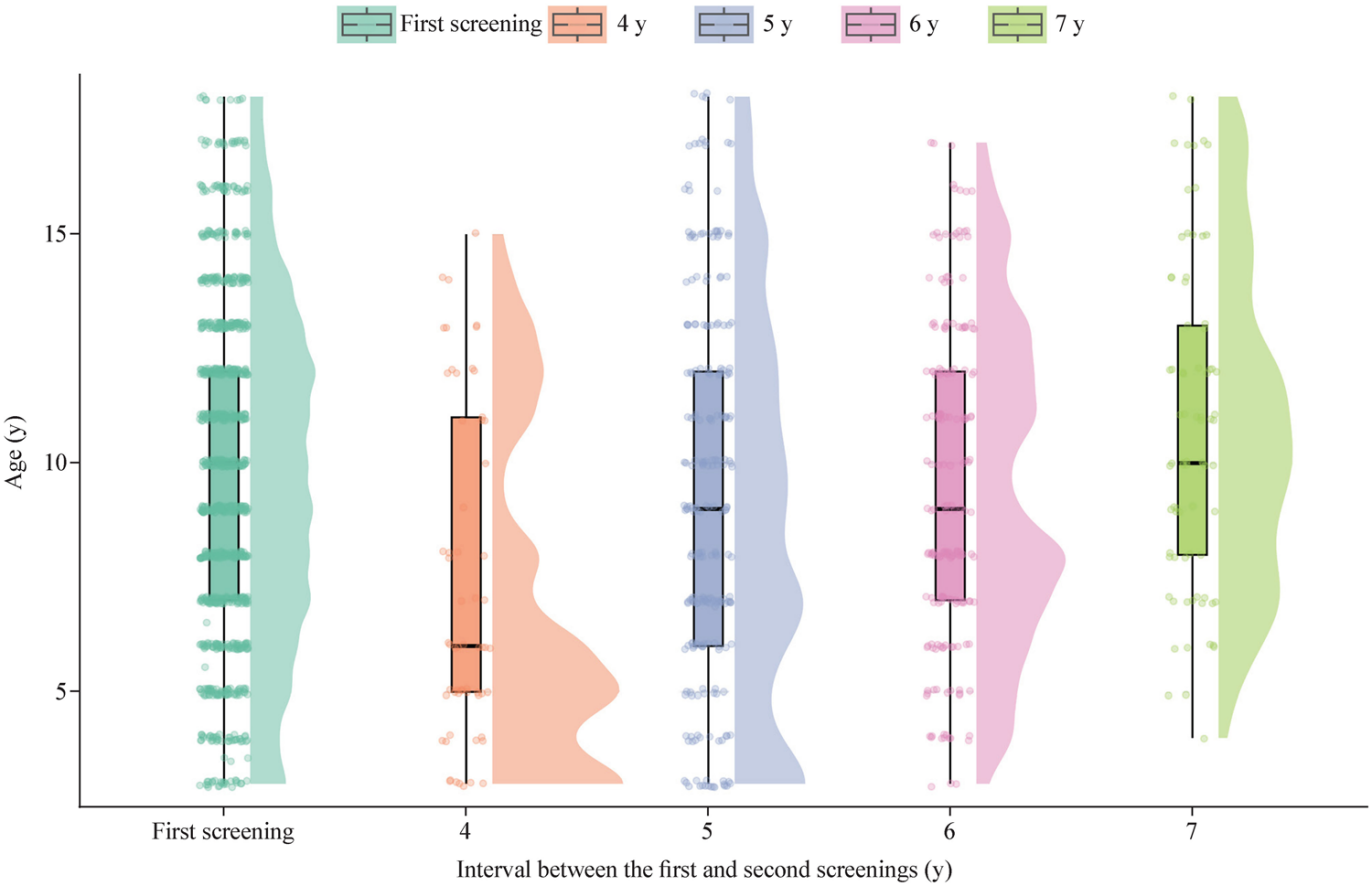
Age distribution of children requiring treatment for congenital heart disease at different screening stages. For each group: box plot (median, interquartile range), scattered dots (individual values), and ridge plot (density distribution) on the right

Significant demographic and clinical differences were observed between children with CHD identified in the first versus second screening round (Supplementary Table 2). These include age (*P* = 0.017), ethnicity (*P* < 0.001), regional type (*P* = 0.017), CHD anatomical complexity (*P* < 0.001), and CHD type distribution (*P* < 0.001). Further stratification of the second screening cohort by time interval since the first screen (4, 5, 6, and 7 years) revealed substantial intergroup heterogeneity (Table 2), Age distribution varied markedly across interval subgroups, with median ages ranging from 6 years in the 4-year interval subgroup to 10 years in the 7-year interval subgroup (Fig. 2). With notable variations in ethnic composition, geographical distribution, and anatomical complexity, there were higher proportions of simple CHD in the interval time subgroups (12.8%–22.3%) compared to the first screen (10.9%, *P* = 0.001). ASD remained the most common CHD type across all groups (34.2%–47.5%).

**Table 2:** Baseline characteristics of children with congenital heart disease requiring treatment identified at different screening stages

Factors	Total	First screening	Second screening (interval time)				*P*
			4 y	5 y	6 y	7 y	
Age	9 (7, 12)	9 (6, 12)	6 (5, 11)	9 (6, 12)	9 (7, 12)	10 (8, 13)	** < 0.001**^**a**^
Sex							0.332^**b**^
Boys	594 (39.7)	385 (41.0)	25 (41.0)	95 (40.6)	61 (33.2)	28 (35.9)	
Girls	901 (60.3)	553 (59.0)	36 (59.0)	139 (59.4)	123 (66.8)	50 (64.1)	
Ethnicity							** < 0.001**^**b**^
Han	736 (49.2)	479 (53.0)	10 (16.4)	156 (66.7)	54 (29.3)	19 (24.4)	
Ethnic minority	759 (50.8)	441 (47.0)	51 (83.6)	78 (33.3)	130 (70.7)	59 (75.6)	
Regional residential type							** < 0.001**^**b**^
Minority autonomous regions	905 (60.5)	546 (58.2)	61 (100.0)	77 (32.9)	143 (77.7)	78 (100.0)	
Non-minority autonomous regions	590 (39.5)	392 (41.8)	0 (0.0)	157 (67.1)	41 (22.3)	0 (0.0)	
Residential altitude (m)							** < 0.001**^**b**^
< 500	109 (7.3)	58 (6.2)	0 (0.0)	0 (0.0)	51 (27.7)	0 (0.0)	
500–1500	788 (52.7)	497 (53.0)	21 (34.4)	77 (32.9)	115 (62.5)	78 (100.0)	
> 1500	598 (40.0)	383 (40.8)	40 (65.6)	157 (67.1)	18 (9.8)	0 (0.0)	
Educational stage							** < 0.001**^**b**^
Kindergarten	333 (22.3)	190 (20.3)	35 (57.4)	60 (25.6)	40 (21.7)	8 (10.3)	
Primary school	808 (54.0)	520 (55.4)	19 (31.1)	118 (50.4)	102 (55.4)	49 (62.8)	
Secondary school	354 (23.7)	128 (24.3)	7 (11.5)	56 (23.9)	42 (22.8)	21 (26.9)	
CHD anatomy							**0.001**^**b**^
Simple	195 (13.0)	102 (10.9)	8 (13.1)	30 (12.8)	41 (22.3)	14 (17.9)	
Moderate complexity	1234 (82.5)	801 (85.4)	51 (83.6)	187 (79.9)	135 (73.4)	60 (76.9)	
Great complexity	66 (4.4)	35 (3.7)	2 (3.3)	17 (7.3)	8 (4.3)	4 (5.1)	
CHD types							** < 0.001**^**b**^
ASD	609 (40.7)	396 (42.2)	29 (47.5)	80 (34.2)	71 (38.6)	33 (42.3)	
VSD	385 (25.8)	265 (28.3)	11 (18.0)	45 (19.2)	49 (26.6)	15 (19.2)	
PDA	259 (17.3)	166 (17.7)	8 (13.1)	47 (20.1)	23 (12.5)	15 (19.2)	
Combined CHD	122 (8.2)	45 (4.8)	7 (11.5)	38 (16.2)	24 (13.0)	8 (10.3)	
Complex CHD	66 (4.4)	35 (3.7)	2 (3.3)	17 (7.3)	8 (4.3)	4 (5.1)	
Other CHD	54 (3.6)	31 (3.3)	4 (6.6)	7 (3.0)	9 (4.9)	3 (3.8)	

Data are presented as median (P25, P75) or *n* (%). Values with *P* < 0.05 are in bold. *CHD* congenital heart disease, *ASD* atrial septal defect, *PDA* patent ductus arteriosus, *VSD* ventricular septal defect. ^a^Kruskal-Wallis *H* test; ^b^Pearson Chi-square test

### Association between screening stage and anatomical complexity of congenital heart disease

The association between screening stage and CHD anatomical complexity using multinomial logistic regression is shown in Table 3. Compared to the first screening, the second screening was associated with significantly lower odds of moderate complexity CHD versus simple CHD across all models [model 3: exp *β* = 0.63, 95% confidence interval (CI) = 0.46–0.86, *P* < 0.01]. No statistically significant association was observed for great complexity versus simple CHD. When stratified by screening interval, children screened after a 6-year interval had significantly lower odds of moderate complexity CHD compared to those in the first screening (model 3: exp *β* = 0.47, 95% CI = 0.30–0.72, *P* < 0.01) (Table 3).

**Table 3:** Associations between screening stage and anatomical complexity in pediatric congenital heart disease

Screening stage	Moderate complexity			Great complexity		
	Model 1	Model 2	Model 3	Model 1	Model 2	Model 3
Binary						
First screening	1.00 (reference)	1.00 (reference)	1.00 (reference)	1.00 (reference)	1.00 (reference)	1.00 (reference)
Second screening	0.59^†^ (0.44, 0.80)	0.64^*^ (0.47, 0.87)	0.63^*^ (0.46, 0.86)	0.97 (0.56, 1.70)	1.06 (0.60, 1.87)	1.03 (0.59, 1.84)
Time interval (y)						
First screening	1.00 (reference)	1.00 (reference)	1.00 (reference)	1.00 (reference)	1.00 (reference)	1.00 (reference)
4	0.81 (0.38, 1.76)	1.21 (0.54, 2.68)	1.29 (0.56, 3.01)	0.73 (0.15, 3.60)	1.13 (0.22, 5.74)	0.96 (0.17, 5.28)
5	0.79 (0.51, 1.23)	0.79 (0.51, 1.24)	0.67 (0.42, 1.06)	1.65 (0.81, 3.35)	1.73 (0.84, 3.57)	1.88 (0.89, 3.95)
6	0.42^†^ (0.28, 0.63)	0.45^†^ (0.29, 0.68)	0.47^†^ (0.30, 0.72)	0.57 (0.24, 1.33)	0.60 (0.25, 1.47)	0.58 (0.24, 1.41)
7	0.55 (0.29, 1.01)	0.54 (0.28, 1.02)	0.65 (0.34, 1.24)	0.83 (0.26, 2.70)	0.75 (0.23, 2.47)	0.70 (0.21, 2.33)

Reference: simple. The analysis involved a multi-model comparison using multinomial logistic regression. Data are presented as exp *β* (95% confidence interval). Model 1: unadjusted model; Model 2: adjusted for sex, age, and ethnicity (Han Chinese or ethnic minority); Model 3: adjusted for sex, age, ethnicity (Han Chinese or ethnic minority), residential altitude, **r**egional residential type (minority autonomous regions or non-minority autonomous regions), and educational stage (kindergarten, primary school, and secondary school). ^*^P < 0.01; ^†^*P* < 0.001

### Characteristics of children with congenital heart disease identified in the second screen by prior screening history

**Fig. 3:**
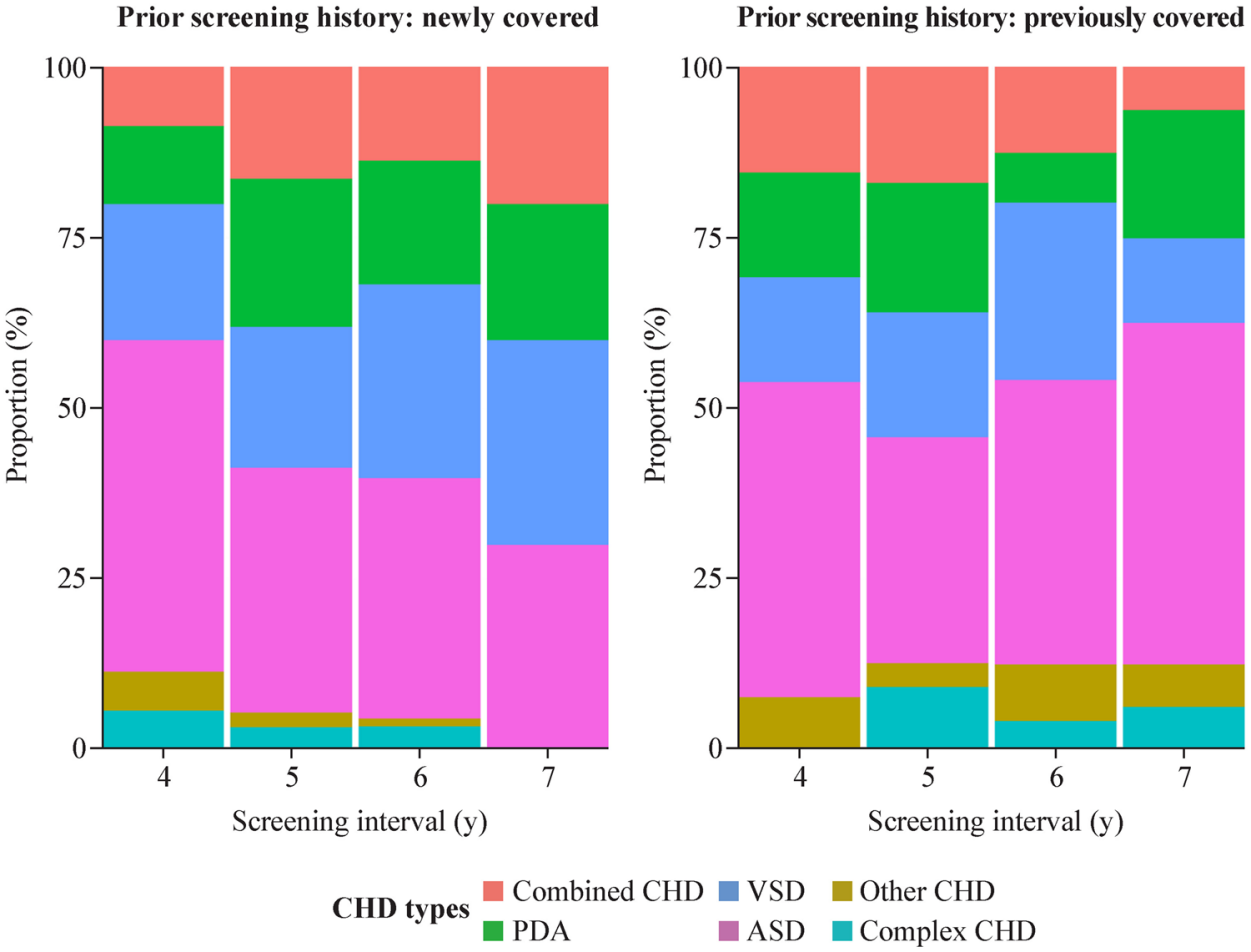
Distribution of CHD types requiring treatment detected at the second screening, stratified by screening interval (4–7 years) and prior screening history (newly covered vs. previously covered). Previously covered: CHD cases that underwent the first screening but were detected only in the second round; newly covered: children who were < 3 years old at the first screening (not age-eligible) and thus entered the screening population for the first time in the second round, and were identified as having CHD. *CHD* congenital heart disease, *ASD* atrial septal defect, *PDA* patent ductus arteriosus, *VSD* ventricular septal defect

**Fig. 4:**
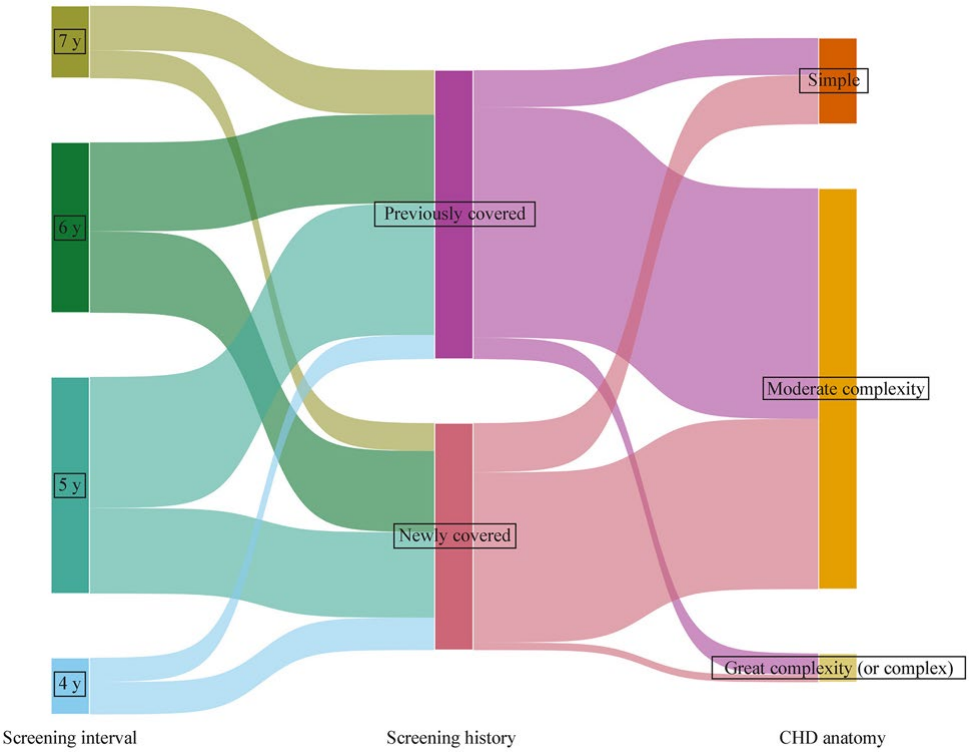
Sankey diagram illustrating epidemiological linkages between interval since first screening, prior screening history, and congenital heart disease (CHD) anatomy in children requiring treatment as identified during the second screening. The first column represents the interval since the first screening, the second column indicates the prior screening history, and the third column displays the CHD anatomy

Supplementary Table 3 compared children with CHD identified within the second screening, stratified by prior screening history. Significant differences were observed in age (median: 6 vs. 12 years, *P* < 0.001), educational stage (*P* < 0.001), CHD anatomical complexity (*P* = 0.004), and screening interval distribution (*P* = 0.035). No statistically significant differences were found in sex, ethnicity, or CHD type distribution between groups. Specifically, anatomically simple CHD was more prevalent in the newly covered group (21.6% vs. 12.8%), whereas great complexity CHD was more common in the previously covered group (7.4% vs. 3.3%) in the second round of screening. ASD remained the most common CHD type in both groups (Figs. 3 and 4).

### Association between prior screening history and anatomical complexity of congenital heart disease detected at the second screen

Within the second screen, newly covered CHD showed significantly lower odds of both moderate complexity (model 3: exp *β* = 0.58, 95% CI = 0.36–0.92, *P* < 0.05) and great complexity (model 3: exp *β* = 0.29, 95% CI = 0.11–0.71, *P* < 0.01) compared to previously covered CHD. These associations remained consistent across all adjusted models (Table 4). In addition, detailed results of the multivariate logistic regression models examining the complex CHD (binary) risk associated with prior screening history are presented in Supplementary Tables 4 and 5. In brief, longer intervals since the first screening (especially 5 and 7 years) were associated with higher detection of complex CHD at the second screening (Supplementary Table 4), and previously covered children showed a trend toward higher risk of complex CHD compared with newly covered children (Supplementary Table 5).

**Table 4:** Association between prior screening history and anatomical complexity in pediatric congenital heart disease detected at second screen

Prior screening history	Moderate complexity			Great complexity		
	Model 1	Model 2	Model 3	Model 1	Model 2	Model 3
Previously covered	1.00 (reference)	1.00 (reference)	1.00 (reference)	1.00 (reference)	1.00 (reference)	1.00 (reference)
Newly covered	0.56^*^ (0.36, 0.88)	0.58^*^ (0.37, 0.92)	0.58^*^ (0.36, 0.92)	0.26^†^ (0.11, 0.65)	0.28^†^ (0.11, 0.69)	0.29^†^ (0.11, 0.71)

Reference: simple. The analysis involved a multi-model comparison using multinomial logistic regression. Data are presented as exp *β* (95% confidence interval). Model 1: unadjusted model; Model 2: adjusted for sex and ethnicity (Han Chinese or ethnic minority); Model 3: adjusted for sex, ethnicity (Han Chinese or ethnic minority), residential altitude, regional residential type (minority autonomous regions or non-minority autonomous regions), time interval since the first screening (4, 5, 6, and 7 years). ^*^P < 0.05; ^†^*P* < 0.01

## Discussion

A key contribution of this study was the identification and characterization of CHD cases missed during initial screening. Children who were screened in the first round but diagnosed only in the second were significantly older and had higher odds of presenting with moderate or great anatomical complexity. This subgroup likely represented false-negative results from the initial auscultation-based screening, which was known to have limited sensitivity for subtle or anatomically atypical lesions. In addition, some complex CHD subtypes may have a delayed clinical presentation, becoming detectable only as children grow older. These findings highlighted that a single CHD screening round, while effective for detecting prevalent and clinically apparent cases, failed to capture a meaningful number of cases that require intervention.

The key finding of a lower overall prevalence of treatable CHD in the second screening round (0.53 per 1000) compared to the first (0.92 per 1000) strongly suggests that an initial comprehensive screen significantly reduces the pool of undiagnosed cases. Notwithstanding this positive finding, alternative sources of bias warrant consideration. First, population mobility between the two screening rounds could have introduced bias, as children who emigrated from the study area were not tracked, whereas those who immigrated may have differed systematically in baseline CHD risk profiles. In Yunnan Province, approximately 2.18% of compulsory education-aged children are not stably enrolled in school (consolidation rate: 97.82%), and over 15 million rural workers have migrated for employment, underscoring the potential scale of mobility-related bias in this study setting [18]. Second, 18 townships/subdistricts that participated in the baseline screening were excluded from the subsequent round owing to logistical constraints. The absence of these areas, which may have differed from the retained areas in terms of CHD prevalence or detection efficiency, may also have introduced bias.  Notably, the prevalence of CHD requiring treatment in both rounds of the present study (0.92 per 1000 and 0.53 per 1000) was lower than the 0.12% (1.2 per 1000) reported in our previous studies from China and Cambodia [19]. Given that a uniform screening methodology was applied, the observed variation may point to substantive differences in factors such as environmental exposures, health education, and improved services over time as potential contributors to the distinct risk profiles identified. [20–22].

The spectrum of CHD requiring treatment in this study (ASD, VSD, and PDA are the most prevalent) was consistent with the established epidemiological profile of CHD reported in large-scale pediatric registries and population-based studies [23–25]. In addition, case characteristics differed significantly between the two screenings. The first screening detected more severe cases (higher anatomical complexity), whereas the second screening—particularly after a 6-year interval—identified milder or previously missed CHD. These findings support the value of periodic, rather than one-time, school-based screening to continuously address the evolving burden of treatable CHD in resource-limited settings [26, 27].

In addition, the stratification of CHD identified within the second screen by the newly covered group and prior screening history further clarifies that the newly covered group consisted entirely of children who had not yet reached school age during the first screening round. The significantly younger age profile and higher proportion of anatomically simple CHD could be largely explained by the fact that they were predominantly composed of children born in or after 2018, a period when systematic neonatal CHD screening had commenced across China [28]. However, neonatal screening coverage was not universal during this period, particularly in rural or under-resourced areas. Thus, the school-based auscultation program served as a complementary safety net. For the “newly covered” group, some missed neonatal screening entirely, while others had false-negative results or developed subtle lesions detectable only with age. As a result, most severe or clinically overt cases were likely identified and managed during the perinatal or infancy periods. This left a residual pool of subtle defects that remained undiagnosed until later detection through school-based screening.

Conversely, children who had been screened previously but remained undiagnosed presented at an older age with a higher proportion of complex defects. This pattern may be explained by several factors. First, false-negative results in the initial screen are likely due to the limited sensitivity of auscultation-based methods in detecting anatomically atypical lesions [29, 30]. Second, according to the AHA classification, “great complexity” includes not only classic cyanotic lesions (e.g., transposition of the great arteries, pulmonary atresia, and truncus arteriosus) but also many acyanotic or mildly symptomatic variants (e.g., crisscross heart, isomerism, heterotaxy syndromes, double-outlet ventricle, and functionally single ventricle variants without early cyanosis). In our missed cohort, these subtle subtypes predominated, including well-balanced tetralogy of Fallot and other complex lesions that presented without typical signs. These subtypes often lack characteristic murmurs or cyanosis in early childhood, making them challenging to detect. Therefore, the missed diagnoses in our study reflect anatomical subtlety [17, 31]. Finally, potential screening coverage gaps and population mobility likely contributed to the detection of previously unscreened children in subsequent screening rounds. Collectively these reinforce the necessity for implementing periodic re-screening programs to effectively identify both incident cases in new birth cohorts and residual, undiagnosed conditions among previously screened children.

Our results provided empirical support for the implementation of systematic repeat screening programs as a necessary component of CHD surveillance in resource-limited regions. The decline in overall prevalence from the first to the second screening should not be misinterpreted as a reduced need for ongoing surveillance. Rather, it reflected the successful clearance of the prevalent case backlog, after which repeat screening serves to identify both new incidents (predominantly in younger, newly covered children) and residual missed cases (often older and with more complex defects). This dual case-finding role ensured that children with CHD were identified at a stage when timely intervention could still improve outcomes, thereby reducing preventable morbidity and mortality.

### Limitations

This study has several limitations. First, the screening protocol relied on cardiac auscultation as the initial step, which is subject to variability in examiner skill and sensitivity [32]. This is particularly so for subtle or hemodynamically balanced lesions. Moreover, this may have contributed to false-negative results in both screening rounds, potentially underestimating the true prevalence of treatable CHD. Second, our classification of “previously covered” children was based on geographic coverage rather than confirmed individual-level screening history, as individual medical records or screening results from the first round were not available. This limited our ability to verify whether these children were actually screened, auscultated, or declared healthy at that time. Therefore, we could only estimate the proportion of cases arising from previously covered populations, without being able to precisely distinguish between potential false negatives, true incident cases, and children who were eligible but not physically screened during the first round. Third, the analysis did not fully account for potential confounders such as socioeconomic factors, maternal health characteristics, or genetic predispositions, which may interact with environmental and ethnic variables to shape CHD risk profiles [33, 34]. Fourth, although the dual-round design cannot determine the optimal screening interval with high precision, it provides important empirical evidence that a 6–7 year interval is clinically meaningful, successfully identifying complex CHD cases that would have otherwise been missed until adulthood. Extended follow-up beyond two rounds would further refine this evidence. Finally, a formal cost–benefit analysis was beyond the scope of this study, as detailed economic data were not collected; future research should evaluate the cost-effectiveness of different rescreening strategies in resource-limited settings.

### Future perspectives

Integration of portable echocardiography into community-based screening protocols holds promise for addressing the inherent limitations of auscultation-dependent approaches. While current strategies reserve echocardiography for cases with positive or equivocal auscultatory findings, we propose a tiered optimization for future programs: (1) universal auscultation as an initial, low-cost filter; (2) targeted portable echocardiography for all individuals with abnormal or indeterminate auscultation, as currently practiced; and (3) low-threshold echocardiographic assessment for high-risk subgroups—including children with syndromic features, family history of CHD, or clinical suspicion of acyanotic complex lesions—even in the absence of an audible murmur. Such an approach would not only reduce the circularity inherent in evaluating “missed cases” but also enhance detection sensitivity for anatomically subtle yet hemodynamically significant defects. Future studies should prospectively evaluate the cost-effectiveness and diagnostic yield of such hybrid screening models in resource-limited settings.

In conclusion, this study underscores the vital public health importance of implementing periodic, school-based screening for CHD requiring treatment in resource-limited settings. Systematic first screening can effectively identify and reduce the backlog of undiagnosed cases within a community. The second round of screening captured both incidents and previously missed, often subtler forms of CHD. Collectively, these findings argue against reliance on one-time screening efforts and provide strong empirical support for establishing structured, periodic screening programs to ensure continuous case detection and timely intervention. This can improve long-term health outcomes for children with CHD in underserved populations.

## Supplementary Information

Below is the link to the electronic supplementary material.

Supplementary file 1 (PDF 231 KB)

## Data Availability

Data that support the findings of this study are available from the corresponding author upon reasonable request.
