# Supplementary material for: Repeat community-based screening for school-age congenital heart disease: impact on the detection of cases requiring treatment in resource-limited settings
Source: World J Pediatr. 2026 Jul 27;22(6):691–701. doi: 10.1007/s12519-026-01060-3 (PMC13433719; doi:10.1007/s12519-026-01060-3)
Supplement: Supplementary file 1 [file 12519_2026_1060_MOESM1_ESM.pdf]

Supplementary materials

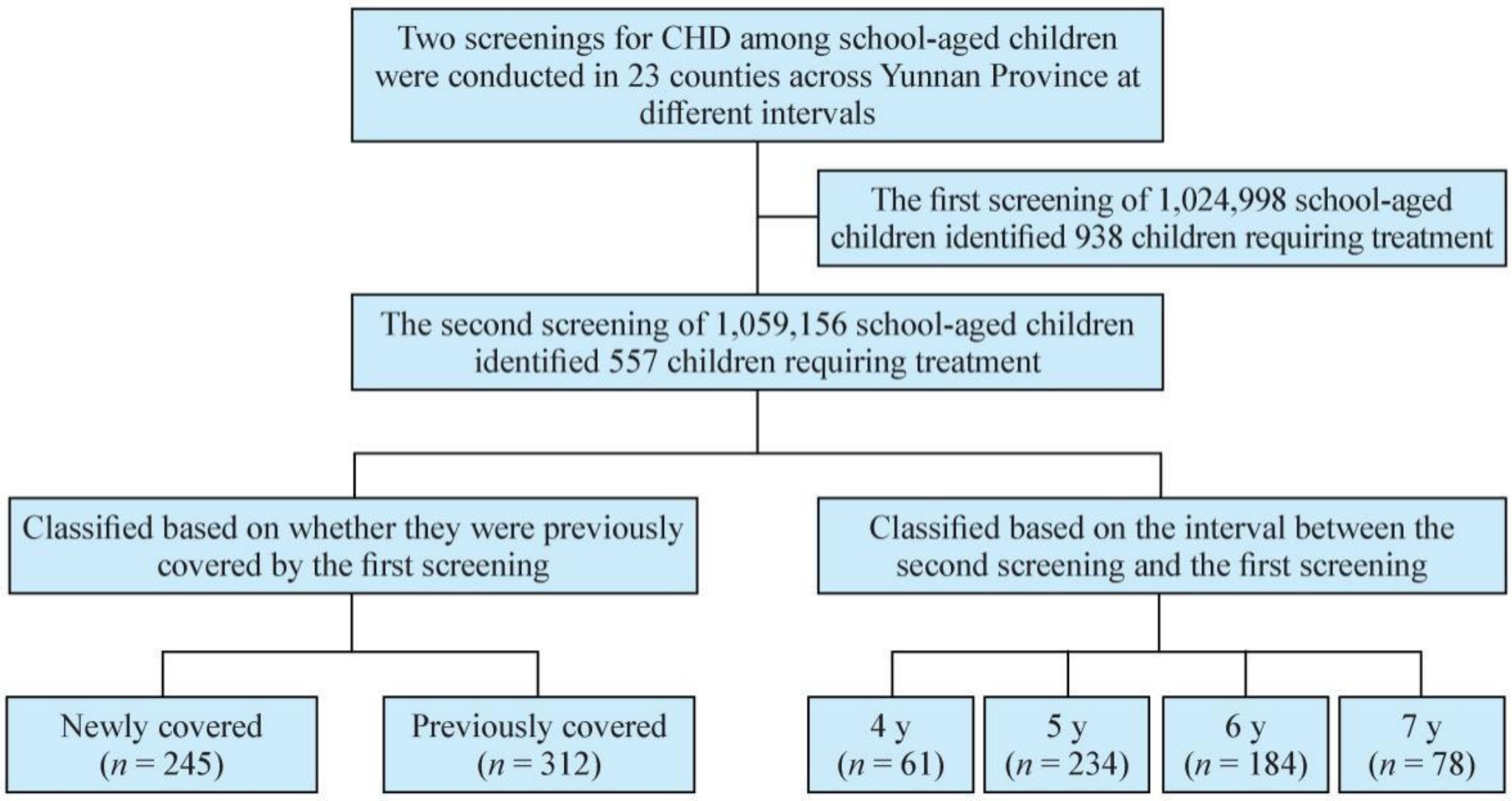

Supplementary Fig. 1 Flowchart of participant selection. *CHD* congenital heart disease

Supplementary Table 1. ICD-10-CM codes for congenital heart (CHD) diseases screened in this pediatric study,

CHD Diagnosis	ICD-10-CM Code
Patent foramen ovale	Q21.101
Atrial septal defect	Q21.100
Ventricular septal defect	Q21.000
Bicuspid aortic valve(BAV)	Q23.101
Patent ductus arteriosus	Q25.000
Tricuspid regurgitation	I07.100
Pulmonary stenosis	Q22.100
Aortic stenosis	Q25.300
Mitral valve prolapse	I34.100
Atrial septal aneurysm	I25.304
Cardiac dilatation	Q24.000
Aneurysm of membranous ventricular septum	I25.300×005
Persistent left superior cava	Q26.100
Dextrocardia	Q24.000
Endocardial cushion defect(ECD)	Q21.207

Supplementary Table 2. Baseline characteristics of children with congenital heart disease identified at different screening stages

Factors	Total	First screening	Second screening	*P*
Age	9 (7, 12)	9 (6, 12)	10 (7, 12)	**0.017**[a]
Sex				0.178[b]
Boys	594 (39.7)	385 (41.0)	209 (37.5)	
Girls	901 (60.3)	553 (59.0)	348 (62.5)	
Ethnicity				**< 0.001**[b]
Han	736 (49.2)	479 (53.0)	239 (49.2)	
Ethnic minority	759 (50.8)	441 (47.0)	318 (57.1)	
Regional type				**0.017**[b]
Minority autonomous regions	905 (60.5)	546 (58.2)	359 (64.5)	
Non-minority autonomous regions	590 (39.5)	392 (41.8)	198 (35.5)	
Altitude (m)				0.095[b]
< 500	109 (7.3)	58 (6.2)	51 (9.2)	
500-1500	788 (52.7)	497 (53.0)	291 (52.2)	
> 1500	598 (40.0)	383 (40.8)	215 (38.6)	
Educational stage				0.052[b]
Kindergarten	333 (22.3)	190 (20.3)	143 (25.7)	
Primary school	808 (54.0)	520 (55.4)	288 (51.7)	
Secondary school	354 (23.7)	128 (24.3)	226 (22.6)	
CHD anatomy				**< 0.001**[b]
Simple	195 (13.0)	102 (10.9)	93 (16.7)	
Moderate complexity	1234 (82.5)	801 (85.4)	433 (77.7)	
Great complexity	66 (4.4)	35 (3.7)	31 (5.6)	
CHD types				**< 0.001**[b]
ASD	609 (40.7)	396 (42.2)	213 (38.2)	
VSD	385 (25.8)	265 (28.3)	120 (21.5)	
PDA	259 (17.3)	166 (17.7)	93 (16.7)	
Combined CHD	122 (8.2)	45 (4.8)	77 (13.8)	
Complex CHD	66 (4.4)	35 (3.7)	31 (5.6)	
Other CHD	54 (3.6)	31 (3.3)	23 (4.1)	

Data are presented as median (P25, P75) or *n* (%). Values with $P < 0.05$ are in bold. *CHD* congenital heart disease, *ASD* atrial septal defect, *PDA* patent ductus arteriosus, *VSD* ventricular septal defect. [a]Kruskal-Wallis *H* test; [b]Pearson Chi-square test

Supplementary Table 3. Characteristics of children with congenital heart disease detected at the second screening, stratified by prior screening history

Factors	Total	Newly covered	Previously covered	*P*
Age	10 (7, 12)	6 (5, 7)	12 (10, 14)	**< 0.001[a]**
Sex				0.155[b]
Boys	209 (37.5)	100 (40.8)	109 (34.9)	
Girls	348 (62.5)	145 (59.2)	203 (65.1)	
Ethnicity				0.219[b]
Han	239 (49.2)	98 (40.0)	141 (45.2)	
Ethnic minority	318 (57.1)	147 (60.0)	171 (54.8)	
Regional type				0.277[b]
Minority autonomous regions	359 (64.5)	164 (66.9)	195 (62.5)	
Non-minority autonomous regions	198 (35.5)	81 (33.1)	117 (37.5)	
Altitude (m)				0.056[b]
< 500	51 (9.2)	19 (7.8)	32 (10.3)	
500-1500	291 (52.2)	142 (58.0)	149 (47.8)	
> 1500	215 (38.6)	84 (34.3)	131 (42.0)	
Educational stage				**< 0.001[b]**
Kindergarten	143 (25.7)	143 (58.4)	0 (0.0)	
Primary school	288 (51.7)	102 (41.6)	186 (59.6)	
Secondary school	226 (22.6)	0 (0.0)	126 (40.4)	
Years since first screening (y)				**0.035**
4	61 (11.0)	35 (14.3)	26 (8.3)	
5	234 (42.0)	92 (37.6)	142 (45.5)	
6	184 (33.0)	88 (35.9)	96 (30.8)	
7	78 (14.0)	30 (12.2)	48 (15.4)	
CHD anatomy				**0.004[b]**
Simple	93 (16.7)	53 (21.6)	40 (12.8)	
Moderate complexity	433 (77.7)	184 (75.1)	249 (79.8)	
Great complexity	31 (5.6)	8 (3.3)	23 (7.4)	
CHD types				0.066[b]
ASD	213 (38.2)	90 (36.7)	123 (39.4)	
VSD	120 (21.5)	60 (24.5)	61 (19.6)	
PDA	93 (16.7)	46 (18.8)	47 (15.1)	
Combined CHD	77 (13.8)	36 (14.7)	43 (13.8)	
Complex CHD	31 (5.6)	8 (3.3)	20 (6.4)	
Other CHD	23 (4.1)	5 (2.0)	18 (5.8)	

Data are presented as median (P25, P75) or *n* (%). Values with $P < 0.05$ are in bold. Previously covered: CHD cases that underwent the first screening but were detected only in the second round; Newly covered: children who were <3 years old at the first screening (not age-eligible) and thus entered the screening population for the first time in the second round, and were identified as having CHD. *CHD* congenital heart disease, *ASD* atrial septal

defect, *PDA* patent ductus arteriosus, *VSD* ventricular septal defect. [a]Kruskal-Wallis *H* test; [b]Pearson Chi-square test

Supplementary Table 4. Association between years since first screening and prior coverage status in children with CHD detected at second screening

Years since first screening	Model 1		Model 2		Model 3	
	OR (95% CI)	*P*	OR (95% CI)	*P*	OR (95% CI)	*P*
4 y	1.00 (reference)		1.00 (reference)		1.00 (reference)	
5 y	2.08 (1.17, 3.68)	**0.012**	2.00 (1.10, 3.66)	**0.024**	2.43 (1.13, 5.19)	**0.023**
6 y	1.47 (0.82, 2.63)	0.197	1.43 (0.79, 2.57)	0.236	1.64 (0.75, 3.58)	0.215
7 y	2.15 (1.09, 4.26)	**0.028**	2.12 (1.07, 4.20)	**0.032**	2.34 (1.09, 5.02)	**0.029**
P for trend	0.338		0.274		0.258	

Data were analyzed using multivariate logistic regression. Model 1: unadjusted model. Model 2: adjusted for sex, ethnicity (Han Chinese or ethnic minority). Model 3: adjusted for sex, ethnicity (Han Chinese or ethnic minority), residential altitude, regional residential type (minority autonomous regions or non-minority autonomous regions), CHD anatomy (simple, moderate complexity and great complexity), CHD types (ASD, VSD, PDA, combined CHD, complex CHD and other CHD). Values with $P < 0.05$ are in bold. *CHD* congenital heart disease, *ASD* atrial septal defect, *PDA* patent ductus arteriosus, *VSD* ventricular septal defect, *OR* odds ratio, *CI* confidence interval

Supplementary Table 5. Risk of complex congenital heart disease by prior screening history in multivariate logistic regression models

Prior screening history	Model 1		Model 2		Model 3	
	OR (95% CI)	*P*	OR (95% CI)	*P*	OR (95% CI)	*P*
Newly covered	1.00 (reference)		1.00 (reference)		1.00 (reference)	
Previously covered	2.36 (1.04, 5.37)	**0.041**	2.30 (1.01, 5.25)	**0.048**	2.23 (0.97, 5.13)	0.058

Model 1: unadjusted model. Model 2: adjusted for sex, ethnicity (Han Chinese or ethnic minority). Model 3: adjusted for sex, ethnicity (Han Chinese or ethnic minority), residential altitude, regional residential type (minority autonomous regions or non-minority autonomous regions), time interval since the first screening (4 years, 5 years, 6 years and 7 years). Values with $P < 0.05$ are in bold. *OR* odds ratio, *CI* confidence interval

Supplementary Table 6. Altitude-stratified analysis of CHD. Data are presented for patients requiring treatment and anatomical complexity

Factors	< 500 m	500-1500 m	> 1500 m	*P*
Prevalence of CHD requiring treatment/1000	0.73 (0.35, 1.31)	0.62 (0.49, 1.03)	0.70 (0.43, 0.96)	0.970[a]
CHD anatomy				**< 0.001[b]**
Simple	19 (17.4)	122 (15.5)	54 (13.0)	
Moderate complexity	82 (75.2)	625 (79.3)	527 (82.5)	
Great complexity	8 (7.4)	41 (5.2)	17 (4.5)	

Data are presented as median (P25, P75) or *n* (%). Values with $P < 0.05$ are in bold. *CHD* congenital heart disease. [a]Kruskal-Wallis *H* test; [b]Pearson Chi-square test

Supplementary Table 7. Association between different residential altitudes and anatomical complexity in pediatric congenital heart disease

Residential altitude	Moderate complexity			Great complexity		
	Model 1	Model 2	Model 3	Model 1	Model 2	Model 3
500-1500 m	1.00 (reference)	1.00 (reference)	1.00 (reference)	1.00 (reference)	1.00 (reference)	1.00 (reference)
< 500 m	0.84 (0.49, 1.44)	0.84 (0.49, 1.45)	1.06 (0.59, 1.89)	1.25 (0.51, 3.08)	1.32 (0.53, 3.28)	1.85 (0.69, 4.97)
> 1500 m	1.91[†] (1.36, 2.68)	1.82[*] (1.23, 2.68)	1.03 (0.57, 1.86)	0.94 (0.49, 1.79)	0.76 (0.37, 1.58)	0.56 (0.17, 1.87)

Reference: simple. Analysis involved a multi-model comparison using multinomial logistic regression. Data are presented as Exp β (95% CI). Model 1: unadjusted model. Model 2: adjusted for sex, age, and ethnicity (Han Chinese or ethnic minority). Model 3: adjusted for sex, age, ethnicity (Han Chinese or ethnic minority), screening stages, regional residential type (minority autonomous regions or non-minority autonomous regions), and educational stage (Kindergarten, primary school, and secondary school). *CI* confidence interval. $^{*}P < 0.01$, $^{\dagger}P < 0.001$
